# Exploiting Reactor Geometry to Manipulate the Properties of Plasma Polymerized Acrylic Acid Films

**DOI:** 10.3390/ma12162597

**Published:** 2019-08-15

**Authors:** Karyn Jarvis, Sally McArthur

**Affiliations:** 1 ANFF-Vic Biointerface Engineering Hub, Faculty of Science, Engineering and Technology, Swinburne University of Technology, Hawthorn, VIC 3122, Australia; 2 Biomedical Manufacturing, CSIRO Manufacturing, Clayton, VIC 3153, Australia

**Keywords:** acrylic acid, ellipsometry, plasma polymerization, X-ray photoelectron spectroscopy (XPS)

## Abstract

A number of different reactor geometries can be used to deposit plasma polymer films containing specific functional groups and result in films with differing properties. Plasma polymerization was carried out in a low-pressure custom-built stainless steel T-shaped reactor using a radio frequency generator. The internal aluminium disk electrode was positioned in two different geometries: parallel and perpendicular to the samples at varying distances to demonstrate the effect of varying the electrode position and distance from the electrode on the properties of plasma polymerized acrylic acid (ppAAc) films. The surface chemistry and film thickness before and after aqueous immersion were analysed via X-ray photoelectron spectroscopy and spectroscopic ellipsometry, respectively. For a perpendicular electrode, the ppAAc film thicknesses and aqueous stability decreased while the COOH/R group concentrations increased as the distance from the electrode increased due to decreased fragmentation. For films deposited at similar distances from the electrode, those deposited with the parallel electrode were thicker, had lower COOH/R group concentrations and greater aqueous stability. These results demonstrate the necessity of having a well characterized plasma reactor to enable the deposition of films with specific properties and how reactor geometry can be exploited to tailor film properties.

## 1. Introduction

Plasma polymerization modifies surfaces via the deposition of a thin film containing specific functional groups. The organic monomer is introduced into the chamber as a vapor, fragmented via radio frequency discharge and deposited onto all surfaces in contact with the plasma [1]. The properties of plasma polymer films, such as chemistry, thickness and stability are conventionally controlled by varying the input monomer/gas, plasma power, monomer flow rate and deposition time. Deposition at lower powers reduces monomer fragmentation, which results in the deposition of relatively intact monomer molecules on the surface; however, these films tend to be less stable in solution due to their low crosslinking [2] and incorporation of low-molecular-weight fragments within the film [3,4,5]. Increasing the power tends to produce more stable films due to higher crosslinking; however, the number of functional groups are typically reduced due to greater monomer fragmentation [6].

The properties of plasma polymerized acrylic acid (ppAAc) can also be influenced by other parameters such as co-polymerization [7,8,9,10,11,12], plasma pulsing [13,14,15,16,17,18] and reactor geometry [3,19,20,21]. Acrylic acid is a commonly used monomer for plasma polymerization and produces a negatively charged carboxylic acid terminated surface. Analysis by X-ray photoelectron spectroscopy (XPS) typically displays the C 1s carboxyl group (COOH/R) at 289.2 eV, with a greater retention at lower deposition power. At higher powers, increased fragmentation of acrylic acid results in lower carboxyl group concentrations [17,22] which, therefore, increases the contribution of C–C, C–O and C=O groups at 285.0, 286.5 and 287.9 eV, respectively [23,24]. Acrylic acid has previously been co-polymerized with hexane [7] or octadiene [8,9,10,11,12] to manipulate the chemistry of ppAAc film. The addition of either monomer decreases the COOH/R group concentration as its contribution to the monomer flow increased. Increasing the octadiene concentration also increased film stability due to increased crosslinking within the film [11]. Retention of COOH/R groups in ppAAc films is typically achieved by either continuous wave (CW) mode at low powers or by pulsing the plasma. COOH/R retention in CW is restricted by the lowest power available, typically 1–2 W, while in pulsed plasmas equivalent powers of 0.1 W or even lower can be achieved. For pulsed plasma films the ratio of time on versus time off, the duty cycle, influences the properties of plasma power films [13]. A number of studies have shown that as the time off is increased, the retention of COOH/R groups increases [13,14,15,16,18]. In comparison to CW at the same power, pulsed plasma also increases the concentration of COOH/R groups [15,17]. Both increasing the time off and pulsing rather than CW, increase the concentration of COOH/R groups due to the reduction in average power which reduces monomer fragmentation.

A number of different reactor configurations can be used to deposit plasma polymers [23,25], which influence the properties of ppAAc films [26]. The position of the substrate within the reactor also influences film properties. Previous work by our group has shown that plasma polymerized allylamine and octadiene films deposited in a T-piece reactor increased in film thickness as the distance from the electrode increased, due to decreasing fragmentation, but did not result in changes in the surface chemistry or swelling. ppAAc films, however, exhibited increasing film swelling and carboxylic acid group concentrations but decreasing film thicknesses, as the distance from the electrode was increased [3]. Several studies have deposited ppAAc films at varying sample distances from the monomer inlet. All showed that the concentration of the COOH/R decreased as the distance from the monomer inlet increased, due to the increasing residence time of acrylic acid molecules in the reactor [19,20,21].

ppAAc films have been used for a number of biomedical applications [27]. Biomedical applications of ppAAc typically require aqueous immersion, thus aqueous film stability and a high density of COOH/R functional groups are both required [28]. Keratinocyte growth was increased by the introduction of COOH/R groups in comparison to the octadiene control. Increasing the COOH/R concentration above 2.3% decreased cell attachment, but it was still greater than the control [9]. The adhesion and spreading of fibroblast cells was also greater on surfaces coated via ppAAc films in comparison to the uncoated polystyrene substrate due to the increase in surface free energy resulting from the COOH/R groups [17,29]. Platelets have been exposed to ppAAc surfaces, indicating surfaces that were less thrombogenic than the uncoated substrate. ppAAc surfaces deposited at lower power resulted in reduced platelet adhesion due to their higher concentrations of COOH/R groups [30].

The aim of this work is to optimize the use of reactor geometry to manipulate the thickness, chemistry and aqueous stability of ppAAc films. The electrode was positioned in parallel and perpendicular configurations and ppAAc films were deposited at a power of 30 W with an acrylic acid flow rate of 5 standard cubic centimetres per minute (sccm) for 20 min. The surface chemistry was analysed via XPS, while the film thickness was determined using spectroscopic ellipsometry. Aqueous stability was investigated by immersing the films in Milli-Q water for 18 h followed by XPS and ellipsometry analysis. We have shown that reactor geometry significantly influences ppAAc film properties, thus demonstrating its potential in tailoring film properties for specific applications.

## 2. Materials and Methods

### 2.1. Materials

Acrylic acid (molecular formula C_3_H_4_O_2_, 99% purity) was purchased from Sigma-Aldrich (Sydney, Australia). Silicon wafer (orientation <1-0-0>, thickness 500–550 µm, single side polished, resistivity 1–10 Ω·cm) was purchased from Micro Materials and Research Consumables Pty Ltd. (Melbourne, Australia).

### 2.2. Plasma Polymerization

**Figure 1:**
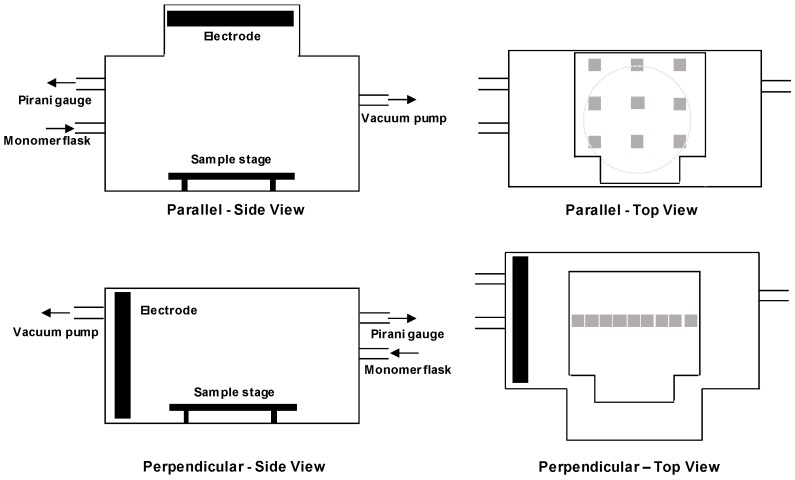
Schematic diagram of side and top views for parallel and perpendicular plasma reactor configurations.

Plasma polymerization was undertaken at low pressure with a stainless steel T-piece reactor with a volume of 16.2 L and 170 mm diameter internal aluminium disk electrode, as previously described [3]. Briefly, the electrode was connected to a 13.56 MHz radio frequency (RF) power source generator (Coaxial Power Ltd., Eastbourne, UK) with an impedance-matching network to generate the plasma. Prior to deposition, the acrylic acid monomer was degassed using three freeze-thaw cycles with liquid nitrogen. No carrier gas was used. In the perpendicular electrode configuration (Figure 1), silicon wafer substrate pieces (approximately 8 mm × 8 mm) were positioned on the sample stage 30–190 mm from the electrode. An aluminum sample stage was used to elevate samples within the reactor chamber to a height of 43.5 mm from the bottom. In the parallel electrode configuration (Figure 1), the electrode was positioned 60, 105 or 140 mm above the sample stage and 9 pieces of silicon wafers were distributed across the samples stage in a 3 × 3 array, as shown in Figure 1. Prior to coating, the chamber was pumped down to a base pressure of at least 1 × 10^−3^ millibar with an Edwards rotary vane RV12 pump. All coatings were deposited for 20 min at a deposition power of 30 W and a flow rate of 5 sccm, which corresponded to a pressure of approximately 2.7 × 10^−2^ mbar. After 20 min, the RF generator was switched off and the monomer allowed to flow for 2 min to allow for complete reaction of any free radicals with the monomer [24]. After the monomer flow was turned off, the reactor was allowed to pump down for a further 2 min to ensure complete evacuation of acrylic acid vapor prior to chamber venting.

### 2.3. Solution Stability

One piece of coated silicon wafer was placed in each well of a 24 well plate with 1 mL of Milli-Q water added to each well. Samples were removed after 18 h, rinsed 3 times with fresh Milli-Q and dried under nitrogen.

### 2.4. X-ray Photoelectron Spectroscopy

XPS spectra of ppAAc-coated silicon wafer pieces before and after solution stability studies were collected using a Kratos Axis Nova instrument (Kratos, Manchester, UK) with a monochromated Al Kα source (source energy 1486.69 eV) at a power of 150 W and a charger neutralizer. Three measurements were taken from each sample with survey spectra collected at a pass energy of 160 eV and a 1 eV step size. High-resolution spectra of C 1s and O 1s were collected at a pass energy of 20 eV and a step size of 0.1 eV. Data was converted to VAMAS format and analysed using CasaXPS software, version 2.3.16. High-resolution C1s spectra from the ppAAc surfaces were curve fitted by applying a Shirley background and Gaussian-broadened Lorentzian functions (30 GL). The full-width-half-maximum of all components was constrained to that of the C–C, C–H peak. The spectra were charge corrected based on the relative position of the C–C, C–H peak to 285 eV.

### 2.5. Spectroscopic Ellipsometry

Ellipsometry measurements were undertaken using an M-2000XI Spectroscopic Ellipsometer (J.A. Woollam Co., Inc., Lincoln, NE, USA). Three measurements were taken from each sample before and after solution stability studies at angles of incidence of 60°, 65°, 70° and 75° at wavelengths of 210–1685 nm. Film thickness was calculated from the acquired data by fitting a B-Spline model on top of a silicon substrate with native oxide layer using CompleteEASE software version 4.92 (J.A. Woollam Co., Inc., USA).

## 3. Results and Discussion

### 3.1. Perpendicular Electrode

**Figure 2:**
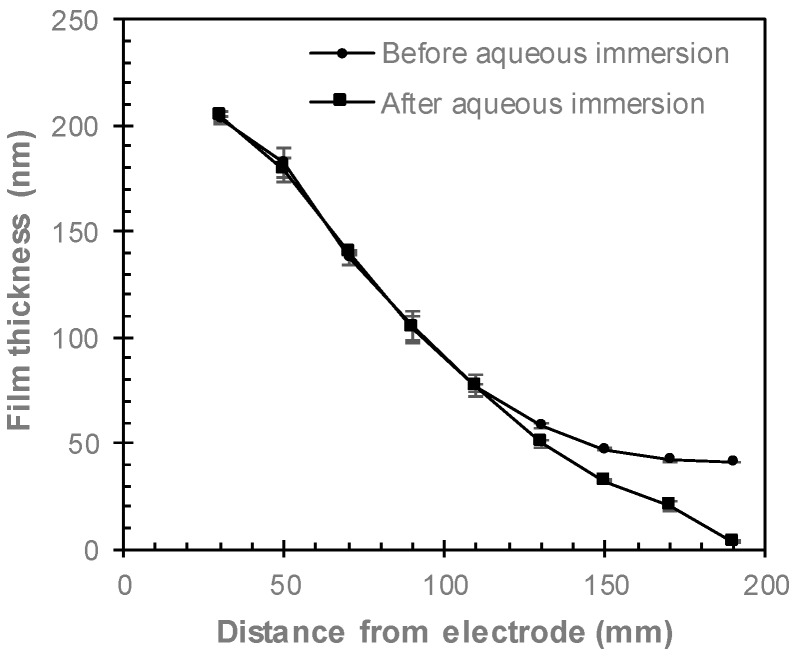
Ellipsometry film thicknesses of ppAAc films before and after immersion in Milli-Q for 18 h as a function of distance from the electrode (error bars ± standard deviation).

ppAAc films were deposited at distances from 30 to 190 mm from the electrode, with their thicknesses shown in Figure 2. Films deposited 30 mm from the electrode had an average thickness of 204 nm, which decreases as the distance from the electrode increases, resulting in average film thicknesses of 42 nm for films deposited at 170 and 190 mm from the electrode. After immersion in Milli-Q for 18 h, films deposited 30 to 110 mm from the electrode did not show any changes in thickness. Films deposited at 130 to 170 mm from the electrode resulted in 15–50% decreases in film thickness, while the film deposited 190 mm from the electrode was almost completely removed by aqueous immersion and only 4 nm thick.

**Figure 3:**
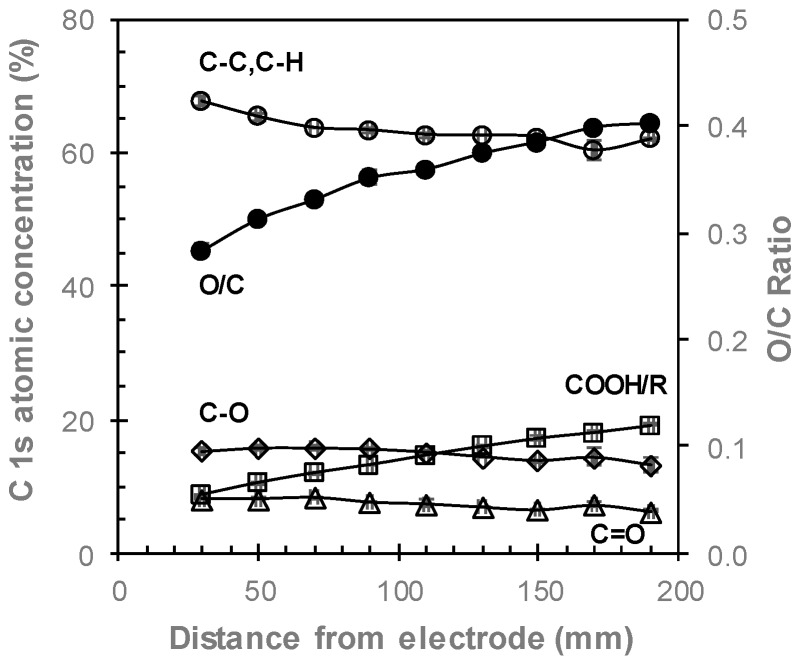
XPS C1s atomic concentrations (○—C–C/C–H, □—COOH/R, ◊—C–O,/C–O–C and ∆—C=O) and O/C ratio (●) for ppAAc films as a function of distance from the electrode (error bars ± standard deviation).

**Figure 4:**
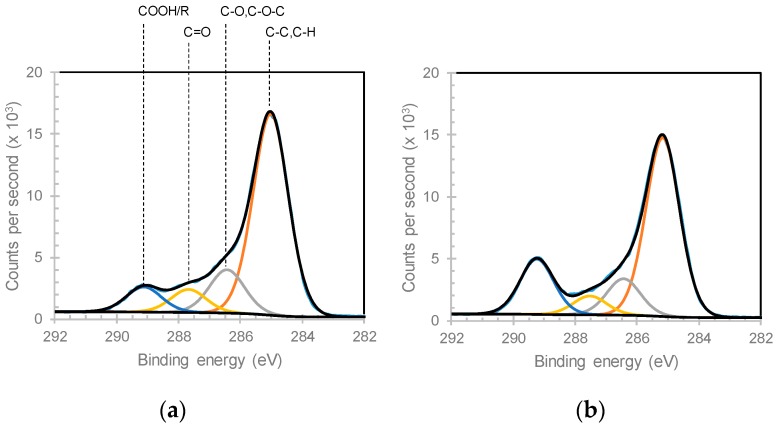
High-resolution C1s XPS spectra for ppAAc films deposited (**a**) 30 and (**b**) 190 mm from the electrode.

The chemistry of ppAAc films as a function of distance from the electrode was investigated via XPS. As XPS cannot detect the presence of hydrogen, XPS reported that the ppAAc films were composed of only carbon and oxygen. The acrylic acid monomer has a O:C ratio of 3:2. The O:C ratio, calculated by dividing the oxygen concentration by the carbon concentration from the survey spectra of ppAAc is lowest for the film deposited 30 mm from the electrode at 0.28 and increases to 0.40 for the film deposited 190 mm from the electrode, as shown in Figure 3. Four component peaks were fitted to the C 1s spectra; hydrocarbon (C–C/C–H, at ~285 eV), alcohols and ethers (C–O/C–O–C at ~286.6 eV), ketones/aldehydes (C=O at ~287.9 eV) and carboxyl and esters (COOH/R at ~289.2 eV) [31], as shown in Figure 4. The COOH/R peak is the combination of contributions from carboxylic acid and ester groups as these cannot be distinguished from one another without derivitization experiments. Derivitization experiments in the literature have, however, shown that the carboxylic acid group contributes to more than 90% of the COOH/R peak [32,33]. The most significant difference in the spectra for ppAAc films deposited at 30 and 190 mm from the electrode is the intensity of the COOH/R group at 289.2 eV. As the distance from the electrode increases, the concentration of C–C/C–H species decreases while the concentration of COOH/R species linearly increase, as shown in Figure 3. The COOH/R concentration increases from 8.7% for a ppAAc film deposited 30 mm from the electrode to 19.2% 190 mm from the electrode. The concentrations of C–O/C–O–C and C=O groups had overall downwards trends as the distance from the electrode increases (Figure 4).

The properties of plasma polymer films are conventionally controlled via the external parameters of plasma power, monomer flow rate and deposition time. In this study, these parameters are kept constant, and therefore, do not influence the film properties. The intrinsic properties of plasma, such as the density of species within the plasma, describe the physical processes taking place within the plasma but are often neglected when describing the deposition of plasma polymer films. The deposition mechanism of plasma polymer films is complex with electrons, radicals, ions and vacuum ultraviolet all potentially playing a role [23]. The decreases in film thickness and increases in the COOH/R groups as the distance from the electrode increases are likely to be contributed to by reduced monomer fragmentation further from the electrode. Our previous research has shown decreasing electron flux with increasing distances from the electrode [3], results in decreased electron collisions [34]. A previous study has shown that the optical emission of an ethylene plasma peaks 10–15 mm from the electrode and decreases as the distance from the electrode increases, indicating decreasing plasma density and therefore lower collisions further from the electrode [35]. Decreased electron collisions will inhibit monomer fragmentation, thus resulting in lower deposition rates and higher COOH/R concentrations. Reduced fragmentation as the distance from the electrode increases the deposition of more intact acrylic acid fragments and thus the greater retention of COOH/R species. Previous studies investigating the influence of plasma power on ppAAc films, attributed greater retention of COOH/R species [17,22,24,32,36] and increases in O/C ratios [19,20,24] at lower powers to reduced acrylic acid fragmentation. The higher concentrations of the C=O and C–O/C–O–C species for ppAAc films deposited closer to the electrode are also attributed to increased fragmentation, where higher concentrations of these species form due to the increased crosslinking resulting [24]. Several studies have shown that as the distance from the monomer inlet increases, the concentration of COOH/R groups and the O/C ratio decrease [19,20,21], due to the residence times of the reactive species that influence COOH retention [34]. As the monomer inlet in the perpendicular electrode configuration is at the opposite end of the reactor to the electrode (Figure 1), our data also shows higher concentrations of COOH/R groups for samples deposited closer to the monomer inlet valve, thus suggesting that the positioning of the monomer inlet also influences the properties of ppAAc films. 

**Figure 5:**
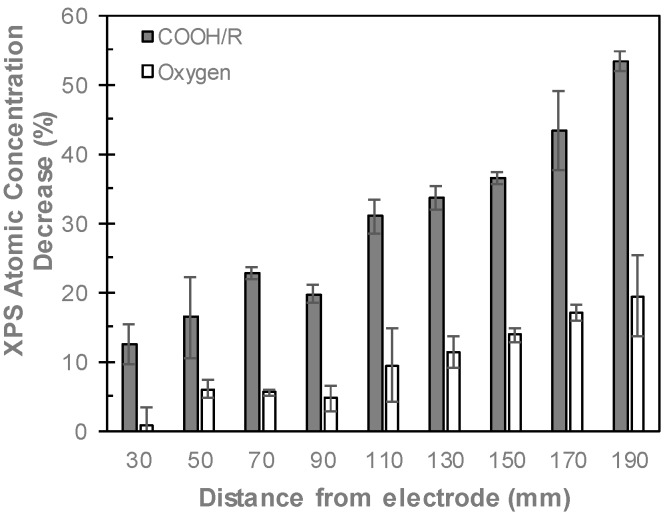
Percentage decrease of XPS COOH/R and oxygen concentrations of ppAAc films after aqueous immersion as a function of distance from the electrode (error bars ± standard deviation).

The decreases in the XPS atomic concentrations of oxygen and COOH/R groups after aqueous immersion are shown in Figure 5. After immersion in Milli-Q for 18 h, the concentrations of both oxygen and COOH/R groups decrease for all films, indicating that any aqueous immersion results in some loss of oxygen. As these films were not washed prior to XPS analysis, small amounts of un-fragmented monomer may be present on the surface, which is washed off via aqueous immersion, thus contributing to the COOH/R loss after aqueous immersion. The COOH/R concentration for the ppAAc film deposited 30 mm from the electrode decreased by 13%, from 8.7% to 7.6%. Such a small increase indicates that the ppAAc film deposited closest to the reactor is relatively stable in aqueous solution. As the distance from the electrode is increased up to 110 mm, the loss of COOH/R increases to up to 30%. These films do not show any reduction in film thickness, as seen in Figure 2, suggesting that these COOH/R groups are lost without any physical film loss. The loss of COOH/R groups are accompanied by losses in the oxygen concentrations, indicating that oxygen-containing species are lost from the surface rather than the COOH/R being oxidized in solution. For ppAAc films deposited 130 to 190 mm from the electrode, where reductions in film thicknesses were observed, the COOH/R loss continued to increase up to 53%.

The lower COOH/R concentrations in the ppAAc films after aqueous immersion likely results from the loss of material from within the film, which has previously been observed in plasma polymerized films [3,4,5], due to decreased fragmentation and, therefore, decreased crosslinking. Increasing the distance from the electrode reduces monomer fragmentation, which also occurs when the plasma power is reduced. Several studies have shown that reduced monomer fragmentation deposits more intact monomer fragments, which decreases aqueous stability [11,20,21,32], while a greater retention of COOH/R has been observed for films deposited at higher powers, where there is greater monomer fragmentation and, therefore, crosslinking [22,32,36]. Such behavior indicates that the ppAAc films deposited at 30–110 mm from the electrode have sufficient crosslinking to resist loss of film thickness, with only small amounts of material from within the films being lost and thus reducing COOH/R loss. ppAAc films deposited at 130–170 mm from the electrode, however, appear to not have sufficient crosslinking to prevent film loss, with the lower crosslinking also resulting in increased COOH/R group losses.

### 3.2. Parallel Electrode

**Figure 6:**
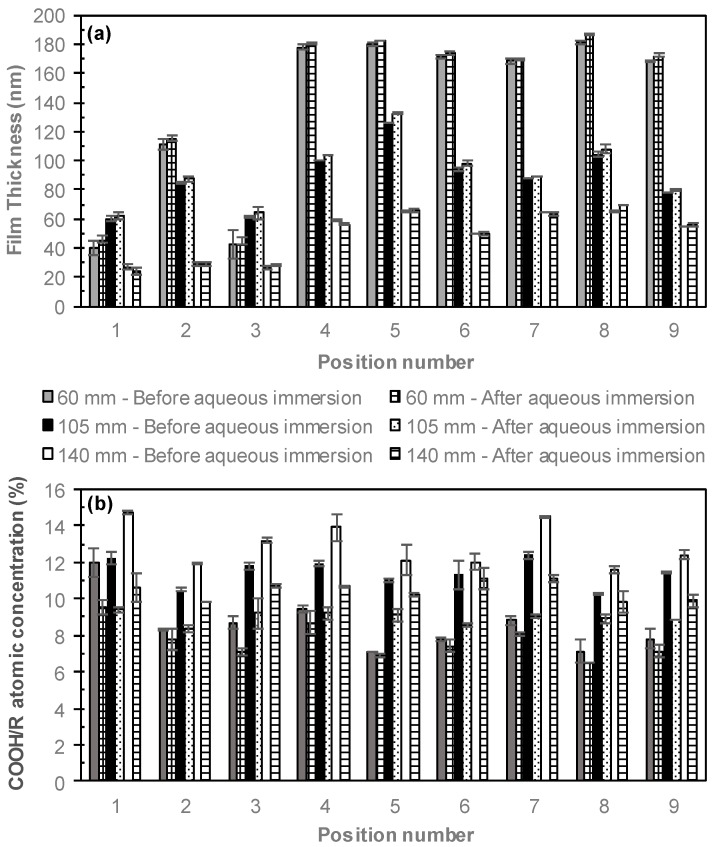
(**a**) Film thickness and (**b**) COOH/R concentrations of ppAAc films deposited 60, 105 and 140 mm from the electrode, before and after aqueous immersion (error bars ± standard deviation).

The orientation of the plasma reactor was modified from a perpendicular to a parallel electrode conformation, as shown in Figure 1. The samples were placed on the sample stage under the electrode, in a 3 × 3 array and labelled with 1 to 9, with 1–3 in the first row, 4–6 in the second row and 7–9 in the last row. The distance between the electrode and the sample stage was varied from 60 to 140 mm. In Figure 6, the film thicknesses and COOH/R concentrations are shown before and after immersion in Milli-Q for 18 h. Increasing the distance from the electrode decreases the film thickness and increases the COOH/R concentrations. The samples situated 60 mm from the electrode had initial thicknesses varying from 40 to 181 nm thick. The greatest variabilities in film thicknesses occurred in the first row, with the film thickness of the samples located in the second and third rows only varying between 168 and 181 nm. The COOH/R concentrations ranged from 7.0% to 11.6%, with typically lower concentrations in the second and third rows than the top row. The lower film thicknesses and higher COOH/R concentrations for the ppAAc films in the top right and top left corner of the top row, were attributed to the electrode position. The electrode is centered over the sample stage and results in regions in the top corners of the sample stage that are not directly under the electrode, as shown in Figure 1, resulting in thinner films with higher COOH/R concentrations. Increasing the distance from the electrode to 105 mm decreased the film thicknesses to 60–126 nm thick, with both the top left and top right samples being considerably thinner. The COOH/R concentrations were higher than for the ppAAc films deposited 60 mm from the electrode, and ranged from 10% to 12%. Increasing the distance further from the electrode to 140 mm resulted in further decreases in the film thickness to 26–65 nm, while the COOH/R concentrations increased to between 11.1% and 14.1%. As expected from the perpendicular electrode configuration, increasing the distance from the electrode decreases the film thickness and increases the COOH/R concentrations due to increased monomer fragmentation and, therefore, deposition rate closer to the electrode with more intact monomer molecules deposited further from the electrode, resulting in higher COOH/R concentrations. The ppAAc films deposited in the upper right and left corners of the stage had thinner films and higher COOH/R concentration due to reduced fragmentation resulting from being situated in the bulk plasma rather than the plasma sheath which results directly under the electrode. Previous studies have shown differences in the properties of plasma films deposited inside compared to outside the plasma sheath [35,37].

After immersion in Milli-Q for 18 h, the COOH/R concentrations of the ppAAc films deposited in the parallel electrode configuration decrease while the film thicknesses slightly increase, which has previously been observed for plasma polymer films after removal from aqueous solution [3] due to incomplete drying resulting in swelling. As the distance from the electrode increases, greater losses of the COOH/R groups after aqueous immersion were again observed. ppAAc films deposited 60 mm from the electrode had COOH/R concentrations of 6–9% after immersion, which corresponded to decreases of 4–9%, excluding the top left sample. At 105 mm and 140 mm from the electrode, COOH/R losses of 15–29% were observed for both. The greater COOH/R losses at greater distances from the electrode are again attributed to reduced monomer fragmentation further from the electrode, which results in less crosslinking and film with reduced solution stability. As no loss of film thickness resulted from films deposited in the parallel electrode configuration, sufficient crosslinking resulted in no physical loss of film but the loss of COOH/R groups suggest the loss of material from within the film.

### 3.3. Comparing Perpendicular and Parallel Electrode Configurations

**Figure 7:**
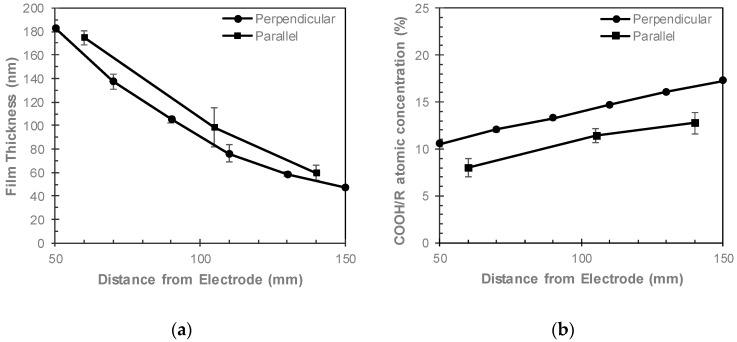
(**a**) Film thickness and (**b**) COOH/R concentrations of ppAAc films deposited in parallel and perpendicular electrode configurations as a function of distance from the electrode (error bars ± standard deviation).

The film thicknesses and COOH/R concentrations of ppAAc films deposited at similar distances from the electrode in perpendicular and parallel configurations prior to aqueous immersion are compared in Figure 7. ppAAc films deposited with the parallel electrode, were approximately 20 nm thicker and had COOH/R concentrations 2–4% lower than those deposited with the perpendicular electrode. These results indicate that the parallel electrode configuration produces greater monomer fragmentation, which results in thicker films and lower concentrations of COOH/R groups, thus indicating electrode orientation also influences monomer fragmentation. Such behavior may be attributed to the plasma sheath, which surrounds the bulk plasma, having reduced electron density in comparison to the bulk plasma [23]. The different electrode orientations in relation to the samples may result in plasma with lower electron density being in contact with the sample surface, thus resulting in lower monomer fragmentation and, therefore, thinner films with higher COOH/R concentrations. After aqueous immersion, the ppAAc films deposited with the parallel electrode demonstrated greater solution stability than those films deposited with the perpendicular electrode. As shown in Figure 2 and Figure 6a, no reduction in film thickness was observed for ppAAc films deposited 140 mm from the parallel electrode, while the ppAAc films deposited 130 mm or more from the perpendicular electrode had reductions in film thickness of 15% or more. Such behavior suggests that the parallel electrode configuration produces films which are more stable in solution, thus indicating greater crosslinking due to greater monomer fragmentation. In comparison to the perpendicular electrode configuration, ppAAc films deposited with the parallel electrode configuration result in films with reduced COOH/R group loss after aqueous immersion. At similar distances from the electrode, ppAAc films deposited with the parallel electrode had COOH/R group losses of 7–22%, while ppAAc films deposited with the perpendicular electrode had COOH/R group losses of 17–38%. The reduced COOH/R group losses for ppAAc films deposited with the parallel rather than perpendicular electrode confirms that in the parallel electrode configuration, greater monomer fragmentation and, therefore, crosslinking occurs. Increased monomer fragmentation results in thinner films with higher COOH/R concentrations and a greater retention of COOH/R groups after aqueous immersion when deposited at similar distances from the electrode. These results highlight the ability to use reactor geometry to deposit ppAAc films with varying thicknesses, COOH/R concentrations and solution stability. 

## 4. Conclusions

Reactor geometry has shown to have a significant effect on the thickness, COOH/R group concentrations and aqueous stability of ppAAc films, and can, therefore, be utilized to produce films with tailored properties. In the perpendicular electrode configuration, as the distance from the electrode increased, the film thicknesses decreased while the COOH/R group concentration increased. Such behavior was attributed to decreased monomer fragmentation as this distance from the electrode increased, which reduced the deposition rate and resulted in the deposition of more intact acrylic acid molecules. After aqueous immersion, the retention of the COOH/R groups decreased as the distance from the electrode was increased, which was proposed to be due to increased fragmentation increasing crosslinking of the ppAAc films deposited closer to the electrode, thus increasing their aqueous stability. In the parallel electrode configuration, the thicknesses and COOH/R group concentrations were relatively uniform as all the substrates were the same distance from the electrode. For ppAAc films that were deposited at similar distances from the electrode, the films deposited with the parallel electrode were thicker, had higher COOH/R concentrations and were more stable after aqueous immersion than those deposited with the perpendicular electrode, thus indicating that the electrode orientation also influences film properties. This study has shown that both the distance from the electrode and the electrode orientation influence the thicknesses, chemistry and aqueous stability of ppAAc films due to monomer fragmentation. These properties, especially COOH/R concentration, are the dictating factors in how such films can be used in applications such as biosensors or biological applications. Precise control over film properties by varying reactor geometry will enable the deposition of films for use in tailored applications.
